# Lysophosphatidic Acid (LPA) and Its Receptors in Mood Regulation: A Systematic Review of the Molecular Mechanisms and Therapeutic Potential

**DOI:** 10.3390/ijms25137440

**Published:** 2024-07-06

**Authors:** Nan Li, Yanchun Li

**Affiliations:** 1 School of Competitive Sports, Beijing Sport University, Beijing 100084, China; ln2023222@163.com; 2 China Institute of Sports and Health Science, Beijing Sport University, Beijing 100084, China; 3 Beijing Key Laboratory of Sports Performance and Skill Assessment, Beijing 100084, China; 4 Key Laboratory for Performance Training & Recovery of General Administration of Sport, Beijing 100084, China

**Keywords:** lysophosphatidic acid (LPA), LPA receptors, gintonin, mood disorders, emotional regulation

## Abstract

Mood disorders affect over 300 million individuals worldwide, often characterized by their chronic and refractory nature, posing significant threats to patient life. There has been a notable increase in mood disorders among American adolescents and young adults, with a rising number of suicide attempts and fatalities, highlighting a growing association between mood disorders and suicidal outcomes. Dysregulation within the neuroimmune–endocrine system is now recognized as one of the fundamental biological mechanisms underlying mood and mood disorders. Lysophosphatidic acid (LPA), a novel mediator of mood behavior, induces anxiety-like and depression-like phenotypes through its receptors LPA1 and LPA5, regulating synaptic neurotransmission and plasticity. Consequently, LPA has garnered substantial interest in the study of mood regulation. This study aimed to elucidate the molecular mechanisms of lysophosphatidic acid and its receptors, along with LPA receptor ligands, in mood regulation and to explore their potential therapeutic efficacy in treating mood disorders. A comprehensive literature search was conducted using the PubMed and Web of Science databases, identifying 208 articles through keyword searches up to June 2024. After excluding duplicates, irrelevant publications, and those restricted by open access limitations, 21 scientific papers were included in this review. The findings indicate that LPA/LPA receptor modulation could be beneficial in treating mood disorders, suggesting that pharmacological agents or gintonin, an extract from ginseng, may serve as effective therapeutic strategies. This study opens new avenues for future research into how lysophosphatidic acid and its receptors, as well as lysophosphatidic acid receptor ligands, influence emotional behavior in animals and humans.

## 1. Introduction

An emotion is a complex psychological state involving three distinct components: a subjective experience, a physiological response, and a behavioral or expressive response [1]. Emotions encompass not only the six basic categories (fear, disgust, anger, surprise, happiness, and sadness) but also include depression, anxiety, and various other emotional states. Each emotional state is associated with specific neural circuits and is significantly influenced by the immune system. Dysregulation of the neuroimmune–endocrine system is now considered one of the fundamental biological mechanisms underlying emotion and mood disorders [1]. Mood disorders affect over 300 million individuals worldwide, often characterized by their chronic and refractory nature, posing significant threats to patient life. There has been a notable increase in mood disorders among American adolescents and young adults, with a rising number of suicide attempts and fatalities, highlighting a growing association between mood disorders and suicidal outcomes [2]. Therefore, emotional regulation is crucial in adapting to environmental changes, and the ability to eliminate abnormal emotional responses is a vital component of a healthy emotional regulation system [3].

Among mood disorders, the incidence of major depressive disorder (MDD) has been increasing annually [4]. According to data from the World Health Organization (WHO), in 2018, MDD ranked third in terms of the disease burden [5]. However, despite its high prevalence, the understanding of its underlying biological mechanisms remains limited. To elucidate the pathogenesis of MDD, the hypothalamic–pituitary–adrenal (HPA) axis dysfunction hypothesis has been proposed [6]. Although this mechanism does not fully explain the pathological basis of MDD, it plays a significant role in guiding the search for new pharmacological treatments, diagnostic criteria, and non-pharmacological preventive measures for MDD [7].

Anxiety disorders are also among the most prevalent emotional disorders globally, encompassing panic disorder (with or without agoraphobia), generalized anxiety disorder, social anxiety disorder, specific phobias, and separation anxiety disorder [8]. These disorders exhibit a high level of comorbidity with other mental disorders. In clinical and epidemiological samples, the comorbidity of anxiety and depression is particularly common [9], with nearly half of individuals diagnosed with depression also meeting the criteria for an anxiety disorder [10]. Moreover, compared to isolated anxiety disorders, this comorbid condition presents a higher incidence rate, an earlier age of onset, and a greater likelihood of recurrence [11], thereby imposing a significant burden on patients. Consequently, further investigation and attention in both research and clinical practice are warranted to develop new therapeutic strategies.

Lysophosphatidic acid (LPA; 1-acyl, 2-hydroxy-sn-glycerol-3-phosphate) is a small signaling glycerophospholipid widely distributed in human tissue, including the cerebrospinal fluid and plasma [12,13,14]. It functions through a complex family of G-protein-coupled receptors (LPARs), which are broadly expressed in the cardiovascular system, immune system, gastrointestinal tract, lungs, endocrine organs, and brain [13,15,16,17,18]. To date, six G-protein-coupled receptors have been identified for the LPAR family, with the LPA1 receptor being particularly relevant to the central nervous system [15]. 

Gintonin, extracted from ginseng, is a novel ligand for G-protein-coupled lysophosphatidic acid (LPA) receptors, consisting of carbohydrates, lipids, and proteins. It triggers intracellular calcium concentration ([Ca^2+^]) transients via the LPA receptor signaling pathway [19]. By stimulating enterochromaffin (EC) cells in the gut in a Ca^2+^-dependent manner, it can induce the release of 5-hydroxytryptamine (5-HT) [20]. Therefore, gintonin can influence 5-HT levels by inducing intracellular [Ca^2+^] transients. Although the effects of gut-derived 5-HT on the central nervous system are not fully understood, previous research indicates that gut 5-HT impacts the vomiting center in the brainstem via the gut afferent system [21,22]. Previous studies have indicated that the plasma serotonin (5-HT) levels decrease in individuals experiencing depression during alcohol withdrawal [23,24]. Nevertheless, the relationship between 5-HT secretion from gut enterochromaffin (EC) cells and the depressive-like behaviors induced by alcohol withdrawal remains unclear. The ginseng extract gintonin is used to improve mood and reduce depressive symptoms. However, the specific active components and the molecular mechanisms responsible for its antidepressant effects remain largely unknown.

The objective of this study is to elucidate the cellular and molecular mechanisms of lysophosphatidic acid (LPA), its receptors, and the LPA receptor ligands in mood regulation. These mechanisms offer significant potential for the development of effective treatments for mood disorders.

## 2. Materials and Methods

**Figure 1:**
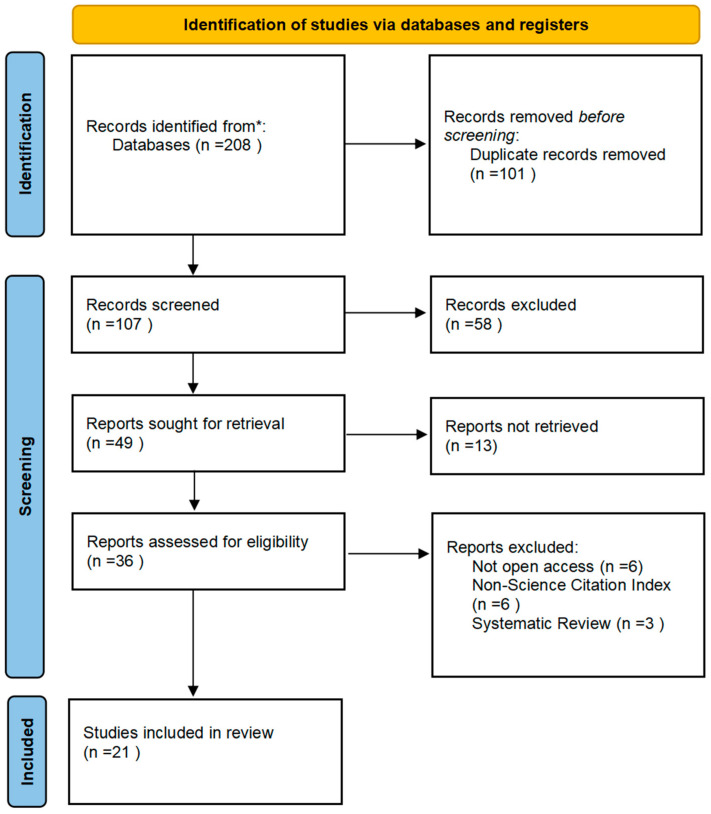
Prisma flow diagram for the selected studies included in the systematic review. * Consider, if feasible to do so, reporting the number of records identified from each database or register searched (rather than the total number across all databases/registers).

The methodology utilized in this systematic review involved the development of search algorithms, selection criteria, and data extraction protocols. Compliance with the Preferred Reporting Items for Systematic Reviews and Meta-Analyses (PRISMA) statement guidelines was maintained, as shown in Figure 1 and detailed in Supplementary Table S1. The search was conducted up to June 2024, concentrating on articles published in English and accessible via online databases such as PubMed (Medline) and Clarivate Web of Science.

Inclusion criteria: Studies presenting data on the relationship between lysophosphatidic acid and mood were considered appropriate for inclusion. The eligible research types included original research articles, clinical trials, randomized controlled trials, and animal studies, all of which were required to be available via open access.

Exclusion criteria: This review excluded publications irrelevant to the specified topic, letters to the editor, brief reports, meta-analyses, systematic reviews, narrative reviews, and papers not written in English.

The search strategy employed the keywords “lysophosphatidic acid”, “mood”, “emotion”, and “depression” and “anxiety”, combined using the Boolean operator “AND”. Articles published in English were screened for eligibility based on their titles and abstracts. Two researchers independently evaluated the articles to eliminate duplicates. Additionally, a manual search was performed by reviewing the reference lists of the identified studies and reviews to locate any relevant studies not captured in the initial search. The complete search strategy is detailed in Table 1.

After searching with the specified keyword combination, 208 articles related to lysophosphatidic acid and mood were identified. After removing duplicates, irrelevant publications, systematic reviews, and papers with open access restrictions, 21 scientifically relevant papers remained for inclusion in this review (Figure 1).

To comprehensively assess the risk of bias in each study, we employed the revised Quality Assessment of Diagnostic Accuracy Studies-2 (QUADAS-2) tool (accessed on June 2024), which encompasses four domains of bias: patient selection, index test, reference standard, and flow and timing [25]. Data were organized and collected using a Microsoft Excel extraction tool in Microsoft Office 2021. Two evaluators (Nan Li and Yanchun Li) independently performed the literature screening and data extraction, cross-checking upon completion. In the event of disagreement between the two evaluators (Nan Li and Yanchun Li), a third evaluator (Min Wu) was consulted for further review (Table 2).

## 3. Results

**Figure 2:**
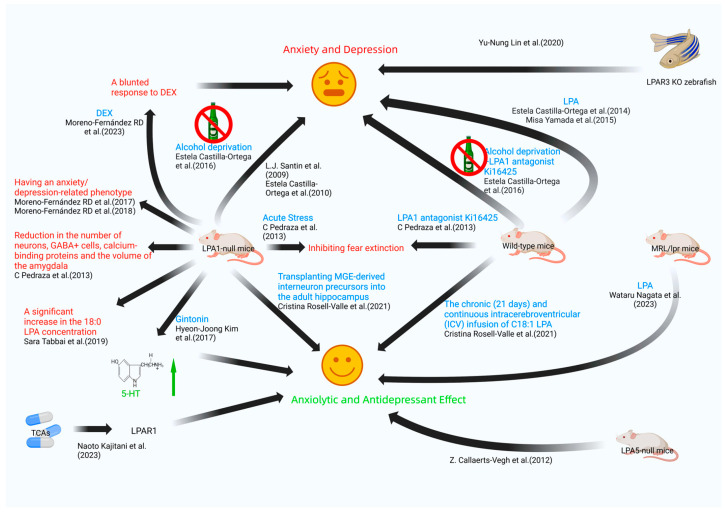
Mechanisms of emotional regulation mediated by lysophosphatidic acid (LPA): animal models and experimental insights into LPA receptors and signaling pathways [26,27,28,29,31,32,33,34,35,37,39,41,42,44,45,46]. DEX: dexamethasone suppression test; LPAR3 KO zebrafish: lysophosphatidic acid receptor 1 knockout zebrafish; 5-HT: 5-hydroxytryptamine; LPAR1: lysophosphatidic acid receptor 1; TCAs: tricyclic and tetracyclic antidepressants.

**Figure 3:**
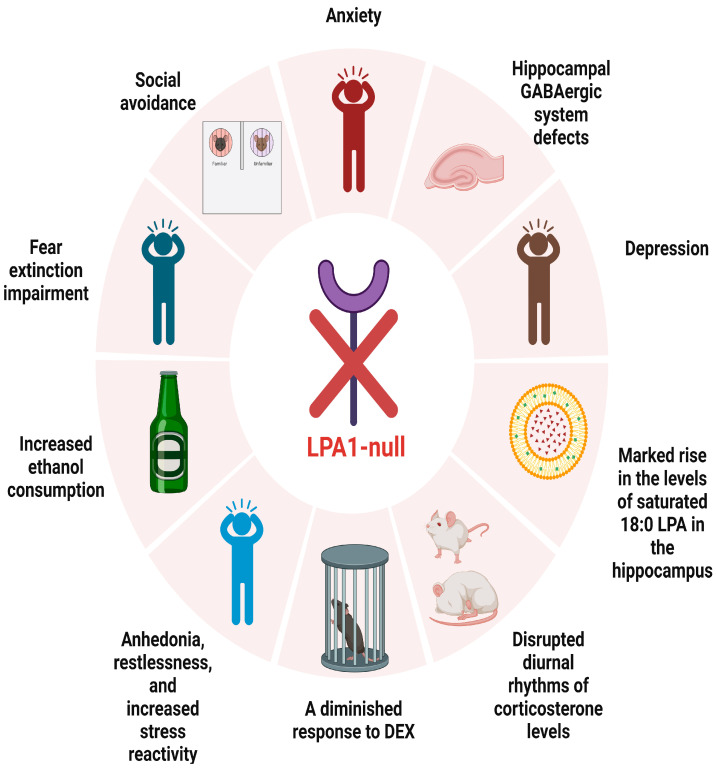
Phenotypic consequences of LPA1-null mutation in mice. LPA: lysophosphatidic acid; DEX: dexamethasone suppression test; LPAR1: lysophosphatidic acid receptor 1.

The 21 studies included in this review cover mood regulation processes related to LPA/LPA receptors and lysophosphatidic acid (LPA) receptor ligand signaling (Figure 2 and Figure 3 and Table 3 and Table 4). Of these studies, 17 are preclinical investigations (Table 3), while four are clinical studies (Table 4). These investigations provide potential pathways for the identification of novel therapeutic strategies to address mood disorders, particularly anxious depression.

In 2009, L.J. Santin et al. [26] examined maLPA1-null mice, revealing significant behavioral anomalies. These mice displayed increased anxiety-like behaviors and reduced exploratory activity in both the open field (OF) and elevated plus maze (EPM) tests. Additionally, maLPA1-null mice exhibited impaired spatial memory in the Morris water maze, showing poor retention and reliance on non-spatial strategies. These results underscore the crucial role of the LPA1 receptor in regulating anxiety and spatial memory.

In 2010, a study by Estela Castilla-Ortega et al. [27] explored the effects of LPA1 receptor deficiency on exploration, anxiety, and spatial memory in mice. The results showed that maLPA1-null mice exhibited significantly reduced exploratory behavior and increased anxiety-like responses in a novel environment compared to wild-type mice. During spatial memory tasks, maLPA1-null mice displayed notable deficits in reference memory, particularly in tasks requiring long-term retention. These mice also showed impaired working memory during initial trials, although their performance improved over time. The study concluded that the LPA1 receptor plays a crucial role in modulating anxiety and spatial memory, highlighting its potential importance in understanding hippocampal-dependent cognitive and emotional processes.

In 2012, Z. Callaerts-Vegh et al. [28] investigated the role of the LPA5 receptor in emotional behaviors using LPA5-null mice. The study revealed that LPA5-null mice exhibited significantly reduced anxiety levels, demonstrating an anxiolytic phenotype. This was demonstrated by an increased number of visits to the center of the arena, reduced thigmotaxis (the tendency to stay close to the walls) in the open field test, and a higher number of entries into the open arms of the elevated plus maze. Interestingly, this anxiolytic phenotype is the opposite to the anxiety-like phenotype observed in LPA1-null mice [26,27]. Specifically, in the brain of the developing mouse, LPA5 expression appears to be diffuse, with higher levels in the prospective forebrain and rostral midbrain regions, whereas LPA1 is predominantly expressed in the rostral forebrain. These findings indicate that the LPA5 receptor plays a crucial role in modulating anxiety and social behaviors, suggesting its potential involvement in emotional regulation and psychopathology.

In 2013, a study by C. Pedraza et al. [29] examined the behavior of LPA1-null mice during fear extinction following foot shock. It was found that fear extinction was notably impaired in the absence of the LPA1 receptor. To replicate the behavioral characteristics of LPA1-null mice, wild-type mice were administered the LPA1 antagonist Ki16425, yielding similar results. These findings indicate that the LPA1 receptor is essential in this emotional process, and its absence or pharmacological inhibition results in significant dysfunction. Additionally, LPA1-null mice displayed morphological abnormalities in the amygdala, such as a decreased volume, reduced neuron count, and lower expression of calcium-binding proteins. This implies that the LPA1 receptor is vital in maintaining the anatomical integrity of the corticolimbic circuits. In summary, this research was the first to elucidate the essential role of the LPA1 receptor in fear conditioning.

In 2014, a study by Estela Castilla-Ortega et al. [30] analyzed the impact of LPA administration on anxiety-like and depression-like behaviors in rats. The findings demonstrated that LPA induced anxiety and depression, underscoring the potential of utilizing the LPA system in pharmacological approaches to treat these conditions. This research was the first to report these findings.

In 2015, Misa Yamada et al. [31] examined the impact of intracerebroventricular LPA administration on adult mouse behavior. The results were consistent with prior studies, showing that LPA induces anxiety-like behavior in mice through the LPA receptors. This indicates that LPA signaling is crucial in modulating anxiety in mice.

In 2016, Estela Castilla-Ortega et al. [32] examined the effects of the genetic deletion or pharmacological blockade of the LPA1 receptor on alcohol consumption. The findings revealed that LPA1-null mice exhibited higher alcohol consumption compared to wild-type mice and showed a markedly increased demand for alcohol following withdrawal. Moreover, LPA1-null mice exhibited heightened anxiety-like behaviors compared to their wild-type counterparts. The acute administration of the LPA1 antagonist Ki16425 led to increased ethanol consumption in wild-type mice but did not influence ethanol consumption in LPA1-null mice. These results suggest that LPA1-null mice could serve as a model for the genetic susceptibility to excessive alcohol consumption and highlight the potential of pharmacologically targeting the LPA/LPA1 receptor signaling pathway to regulate alcohol intake.

In 2017, Moreno-Fernández RD et al. [33] investigated the role of the LPA1 receptor in emotional regulation. The authors first examined the hedonic and despair-like behaviors of wild-type and LPA1-null mice, finding that LPA1-null mice exhibited panic-like responses. They then evaluated the c-Fos levels to gauge the functional activity and constructed brain activation maps using inter-regional correlation matrices. The results indicated that LPA1-null mice displayed anhedonia, restlessness, and increased stress reactivity, behaviors closely associated with the psychopathic endophenotype of depression with anxiety traits. Additionally, LPA1-null mice showed the heightened activation of the limbic system, mirroring observations in patients with depression. After antidepressant treatment, the behavior of LPA1-null mice improved, and brain function normalized. Finally, based on the validity criteria, the study suggested that LPA1-null mice could serve as a viable animal model for the investigation of anxious depression. The findings emphasized the critical role of the LPA1 receptor in emotions, suggesting that its downregulation might be involved in the onset of primary symptoms of depression with anxiety, affecting brain circuits related to reward and emotional regulation. This research was the first work to identify a potential link between the LPA1 receptor and anxious depression, revealing the previously unknown neurobiological underpinnings of this depression subtype. It also opens new avenues for the investigation of therapeutic targets for mood disorders, particularly anxious depression.

In the same year, Hyeon-Joong Kim et al. [34] investigated the effects of gintonin on the enterochromaffin (EC) cell line BON cells and C57BL/6 mice. The investigation revealed that treating BON cells with gintonin induced the release of 5-hydroxytryptamine (5-HT) via LPA receptor-mediated calcium transients ([Ca^2+^]). Additionally, the oral administration of gintonin in mice increased their plasma 5-HT concentrations proportionally with the dose and duration. Given that intestinal enterochromaffin (EC) cells supply most plasma 5-HT, this indicates that oral gintonin may similarly trigger 5-HT release from mouse EC cells, resulting in increased plasma 5-HT levels. Moreover, oral gintonin reduced the immobility time in both the forced swim test (FST) and tail suspension test (TST) during alcohol withdrawal, suggesting its potential to mitigate depressive-like behaviors in mice under these conditions. These findings imply that gintonin promotes 5-HT release in the gut, thereby elevating the plasma 5-HT levels and influencing depression-related behaviors in mice.

In 2018, a study by Moreno-Fernández RD et al. [35] contrasted the behavioral outcomes and brain functional activity of LPA1-null mice with those of mice administered the LPA1 antagonist Ki16425. The results of this study corroborated earlier conclusions that LPA1-null mice serve as a valid animal model for anxious depression, affirming previous experimental data [33]. Nevertheless, it is important to note that the antagonist reproduced only certain effects of LPA1 receptor gene deletion.

Also in 2018, Eun Young Kim et al. [36] conducted a serum lipidomic analysis to discover biomarkers for major depressive disorder (MDD) in drug-free patients. Using liquid chromatography–mass spectrometry, the study identified 37 differentially regulated lipids (DRLs) in the serum of patients with current MDD (cMDD), remitted MDD (rMDD), and healthy controls. The analysis revealed that specific lipid species, such as LPA (16:1) and various triglycerides (TG), could distinguish between these groups with notable accuracy. LPA (16:1), TG (44:0), and TG (54:8) differentiated cMDD patients from controls with 76% accuracy. These findings suggest potential peripheral lipid biomarkers for MDD, emphasizing the role of lipidomics in understanding the pathophysiology and diagnosis of depression.

In 2019, a study by Sara Tabbai et al. [37] was the first to detect, identify, and analyze LPA species in the hippocampi of both LPA1-null and wild-type mice. Moreover, it was pioneering in examining the effect of acute stress on hippocampal LPA species concentrations and their potential relationship with behavior. The results indicated that although the absence of the LPA1 receptor did not change the amounts of LPA species, it did slightly modify their characteristics, notably causing a marked rise in the levels of saturated 18:0 LPA in the hippocampus. Acute stress caused substantial changes in the hippocampal LPA levels, indicating that the hippocampus may be a crucial target for stress-induced alterations in LPA. Finally, intrinsic emotional stress in the elevated plus maze (EPM) altered the LPA profile, revealing associations between specific LPA species and behavioral outcomes. Specifically, the hippocampal 16:0 LPA levels were linked to anxiety, the 18:1 and 18:2 LPA levels correlated with fear, and the 18:0 LPA levels were associated with locomotion. These findings suggest that hippocampal LPA could serve as a critical pharmacological target to mitigate the impact of stress on the hippocampus and emotional behavior.

Also in 2019, Leo Gotoh et al. [38] examined the lysophosphatidic acid (LPA) levels in cerebrospinal fluid (CSF) and plasma samples from patients with major depressive disorder (MDD). The study involved 52 MDD patients and 49 healthy controls for the CSF analysis and 47 MDD patients and 44 controls for the plasma analysis. The results indicated no significant differences in the LPA levels between MDD patients and healthy controls in either CSF or plasma samples. Additionally, there was no correlation between the LPA levels and the severity of the MDD symptoms, as measured by the Hamilton Depression Rating Scale. These findings suggest that the LPA levels in the CSF and plasma are unlikely to serve as biomarkers for MDD diagnosis or severity assessment.

The amino acid sequences of genes related to LPA in zebrafish exhibit 50–70% similarity with those of their mammalian counterparts. In 2020, a study by Yu-Nung Lin et al. [39] conducted extensive behavioral testing on LPAR3 knockout (KO) zebrafish. The study revealed that the LPAR3 KO zebrafish displayed abnormal exploratory behavior and relatively high levels of anxiety. This suggests that the LPA3 receptor may play a role in specific anxiety-related neural circuits. This finding has charted a new course for future research into how the LPA3 receptor and other LPA receptors (LPARs) influence behavior in both rodents and humans.

Also in 2020, Sumaia Riya et al. [40] conducted a study evaluating the serum levels of lysophosphatidic acid (LPA) and lysophosphatidylcholine (LPC) in patients with major depressive disorder (MDD). The study included 53 MDD patients and 50 healthy controls (HCs) and used enzyme-linked immunosorbent assay kits to measure the serum LPA and LPC levels. The results indicated no significant differences in the serum LPA and LPC levels between MDD patients and HCs. Furthermore, there was no significant correlation between these lipid levels and the severity of depression as measured by the Hamilton Depression Rating Scale. The study concluded that the serum LPA and LPC levels are unlikely to serve as biomarkers for MDD.

Previous research has demonstrated that lysophosphatidic acid (LPA) is involved in numerous biological and behavioral processes through its receptors, including hippocampal-dependent memory, adult hippocampal neurogenesis, and emotional regulation. However, most analyses have focused on the effects of acute treatment, and there is a lack of information on the impact of the chronic pharmacological modulation of the LPA/LPA receptor signaling pathway. In this context, in 2021, a study by Cristina Rosell-Valle et al. [41] examined the impact of a 21-day sustained intracerebroventricular (ICV) infusion of C18:1 LPA and the LPA1-3 receptor antagonist Ki16425 on mouse behavior and hippocampal neurogenesis. The results showed that the chronic ICV injection of 18:1 LPA increased the exploratory behavior and reduced anxious tendencies in mice. The long-term pharmacological manipulation of the LPA receptors resulted in altered hippocampal function in the mice. This study found that the prolonged pharmacological intervention involving the LPA receptors impacted emotional regulation, exploratory behavior, and spatial working memory. These changes were accompanied by alterations in hippocampal neurogenesis; neuronal activation in the CA1, CA3, and DG hippocampal subregions; and variations in α-CaMKII and phospho-α-CaMKII levels. These results reinforce earlier findings suggesting that LPA signaling, particularly through the LPA1 receptor, is a susceptibility factor for anxiety and depression in cognitive and emotional regulation [33,35]. Additionally, the LPA receptors may represent potential targets for the development of new antidepressants and anxiolytics.

Previous studies have shown that adult LPA1-null mice exhibit defects in the hippocampal GABAergic system, leading to neuronal and behavioral deficits. This highlights the significance of the LPA1 receptor in these processes. In 2021, a study conducted by Cristina Rosell-Valle et al. [42] examined GABAergic interneurons in the hippocampi of both wild-type and LPA1-null mice. Their analysis revealed that adult LPA1-null mice exhibited a decreased number of hippocampal neurons expressing GABA, calcium-binding proteins (such as parvalbumin, calbindin D28K, and calretinin), and neuropeptides (including neuropeptide Y and somatostatin). Therefore, after initially identifying hippocampal GABAergic system defects in LPA1-null mice, the study observed that transplanting GABAergic precursor cells into the adult hippocampi of these mice tended to normalize or mitigate the cellular and behavioral defects. These results advocate for the application of cell-based therapies in treating brain diseases and propose that the LPA1 receptor may serve as a promising target for neuropsychiatric disorders.

Also in 2021, Wataru Omori et al. [43] conducted a study investigating the cerebrospinal fluid (CSF) levels of lysophosphatidic acid docosahexaenoic acid (LPA 22:6) in patients with major depressive disorder (MDD) and schizophrenia (SCZ). The results demonstrated significantly lower levels of LPA 22:6 in the CSF of patients with MDD and SCZ compared to healthy controls. These reduced levels of LPA 22:6 were negatively correlated with several clinical symptom scores in MDD patients but not in SCZ patients. The findings suggest an abnormality in LPA 22:6 metabolism in these psychiatric conditions and highlight its potential role in the pathophysiology of MDD.

In 2023, a study by Moreno-Fernández RD et al. [44] examined the social behavior of wild-type and LPA1-null mice and conducted the dexamethasone suppression test (DEX). The results showed that LPA1-null mice displayed social avoidance, a diminished response to DEX, and disrupted diurnal rhythms of corticosterone levels. These findings demonstrate that LPA1-null mice serve as an effective animal model for anxious depression, reinforcing previous evidence of the LPA1 receptor’s role in the development of depression-like characteristics and expanding the opportunities for the development of LPA1-receptor-targeted drugs, which may be beneficial in treating mood disorders.

In 2023, a study by Wataru Nagata et al. [45] examined the effects of LPA treatment on microglial activation, impaired neurogenesis, and abnormal behavior in MRL/lpr mice. The study demonstrated that behaviorally abnormal MRL/lpr mice, compared to the control group, exhibited increased levels of CD68, TMEM119 (a specific microglial marker) [22], GFAP, and cleaved caspase-3 in the hippocampus and prefrontal cortex. LPA treatment reduced the expression of CD68, TMEM119, GFAP, and cleaved caspase-3 in MRL/lpr mice and improved their abnormal behavior. Additionally, LPA treatment suppressed the expression of IL-1β and TNF-α in the hippocampal tissue of rats. These findings suggest that LPA treatment may alleviate depressive-like behaviors in MRL/lpr mice by inhibiting microglial activation and reducing blood–brain barrier permeability.

In 2023, a study by Naoto Kajitani et al. [46] hypothesized that the LPA1 receptor could serve as a target for classical antidepressants, including tricyclic and tetracyclic antidepressants (TCAs). Upon activation by TCAs, this receptor influences emotional behavior. The findings indicated that TCAs directly bind to the LPA1 receptor and exhibit G-protein-biased agonism, potentially explaining their antidepressant effects. The unique agonistic activity of the LPA1 receptor is exclusive to TCAs.

The findings from all studies indicate that targeting the LPA/LPA receptor system or employing the ginseng extract gintonin may provide therapeutic benefits for the treatment of mood disorders. However, the precise roles of the LPA3 and LPA5 receptors remain unclear and require further investigation.

## 4. Discussion

This review examined the role of lysophosphatidic acid (LPA) and its receptors, as well as LPA receptor ligands, in mood regulation. The results indicated that wild-type mice exhibited anxiety-like behavior following acute LPA treatment. However, chronic continuous LPA treatment reduced the anxiety-like behavior in these mice. LPA1-null mice also exhibited anxiety-like behavior, whereas LPA5-null mice displayed anxiolytic-like behavior. LPAR3 KO zebrafish exhibited heightened anxiety-like behavior. Gintonin can influence the 5-HT concentrations by inducing intracellular calcium [Ca^2+^] transients, thereby improving mood and alleviating depressive symptoms. These findings underscore the potential of drugs targeting the LPA/LPA receptor system and the ginseng extract gintonin as effective pharmacological approaches for the treatment of mood disorders. In human studies, the relationship between the LPA levels and major depressive disorder (MDD) remains unclear, necessitating further research to validate these associations. These results pave the way for future research into the mechanisms through which lysophosphatidic acid and its receptors, as well as LPA receptor ligands, influence emotional behavior in both animals and humans.

The amygdala, a key site in the corticolimbic circuit, is essential for emotion processing. Impairment in this region can hinder emotional regulation, particularly affecting the fear extinction and stress responses, which are central aspects of anxiety disorders [47,48]. C. Pedraza et al. [29] identified morphological abnormalities in the amygdalae of LPA1-null mice, including a reduced volume, fewer neurons, and the altered expression of calcium-binding proteins. Given the presence of LPA1 receptors in the amygdala, similar to their distribution in the cortex and hippocampus, their prolonged absence could result in specific morphological abnormalities and compromised inhibitory mechanisms, potentially leading to an exaggerated amygdala response to emotional stimuli. It is well known that stimulating the amygdala promotes the release of glucocorticoids [49,50]. Consequently, increased amygdala activity typically results in heightened fear expression [51]. Therefore, a plausible explanation for the impaired fear extinction in LPA1-null mice is that the morphological abnormalities of the amygdala, combined with the enhanced amygdala activity during emotional processing, lead to heightened emotional responses [52]. Consistent with data observed in mice with a reduced amygdala volume in previous studies [53], the corticosterone levels significantly increased, and the amygdala was notably activated after acute stress in these mice. From these findings, we conclude that LPA1-null mice have distinct amygdala mechanisms compared to normal mice. Estela Castilla-Ortega et al. [30] found that although LPA1-null mice displayed anxiety-like phenotypes, wild-type mice also displayed anxiety-like phenotypes after LPA administration, possibly due to LPA1 receptor stimulation, thus presenting contradictory findings. These results support our hypothesis that the development of LPA1-null mice may differ from that of normal mice, with enhanced amygdala activity during emotional processing, as the LPA1 receptor is essential for brain development. [54]. Cristina Rosell-Valle et al. [42] observed that adult LPA1-null mice exhibited a reduced number of hippocampal neurons expressing GABA, calcium-binding proteins (parvalbumin, calbindin D28K, and calretinin), and neuropeptides (neuropeptide Y and somatostatin). Reduced inhibitory control by the GABAergic system in the amygdala may be a common mechanism behind enhanced emotional responses [55]. Additionally, the reduction in calcium-binding proteins in the amygdala could contribute to these abnormal emotional responses. This reduction in calcium-binding proteins may partially explain the abnormal emotional responses observed in LPA1-null mice. c-Fos expression is used as a marker of neuronal activity. Moreno-Fernández RD et al. [33] reported elevated c-Fos levels in the CeA and CA3 regions of LPA1-null mice compared to wild-type controls. However, the molecular mechanisms driving these changes in c-Fos expression in LPA1-null mice remain unclear. All of these data indicate that the absence of the LPA1 receptor induces enhanced amygdala activity during emotional processing, leading to heightened emotional responses.

Furthermore, the prolonged absence of the LPA1 receptor may lead to unknown neuroadaptive responses, including the abnormal regulation of other LPA receptors. This emphasizes the necessity of pharmacological research to elucidate the functions of LPA and its receptors in emotional and behavioral processes, as pharmacological modulation in normal animals might produce distinct outcomes compared to studies in LPA1-null mice. While the role of the LPA1 receptor in neural mechanisms appears probable, the involvement of other LPA receptors in explaining the results observed in LPA1-null mice cannot be ruled out. In 2012, Z. Callaerts-Vegh et al. [28] discovered that mice lacking the LPA5 receptor displayed an anxiolytic phenotype, contrasting with the anxiety-like behaviors observed in LPA1-null mice [26,27]. The differences observed between the LPA1 and LPA5 receptors could be attributed to their distinct expression patterns during neural development [56]. In the developing mouse brain, LPA5 receptor expression is widespread, with elevated levels in the forebrain and rostral midbrain regions [56], whereas LPA1 receptor expression is concentrated in the rostral forebrain and rostral midbrain regions [57]. Therefore, the expression of the LPA5 receptor in the adult brain warrants further investigation. The LPA2-4 receptors are expressed in the adult brain, albeit at lower levels compared to the LPA1 receptor [13]. Currently, no data support the involvement of the LPA2, LPA4, and LPA6 receptors in behavior. The study by Yu-Nung Lin et al. [39] found that LPAR3 gene KO zebrafish displayed reduced social behavior, consistent with the social behaviors observed in LPA5-null mice [54], both exhibiting significantly reduced social exploration behavior when encountering new conspecifics. Additionally, zebrafish are generally diurnal animals, exhibiting increased movement during daylight hours and rest-like behavior after dark. However, in this study, LPAR3 KO zebrafish exhibited the significant dysregulation of locomotor activity across the circadian cycle, with increased activity at night and reduced activity during the day. This observation aligns with previous findings where LPA5-null mice displayed nocturnal hyperactivity in the cage activity test [28]. Furthermore, the researchers observed a consistent decline in norepinephrine and epinephrine levels in the brain and body tissue of LPAR3 KO zebrafish. Low norepinephrine levels are associated with depression and a lack of motivation in animals [58], whereas epinephrine is linked to various emotional responses under stress [59]. Therefore, the aggression and reduced social interaction observed in LPAR3 KO zebrafish may be partly related to the decreased levels of norepinephrine and epinephrine. Furthermore, the serotonin levels were found to be slightly elevated, which may have contributed to the behavioral changes observed in the LPAR3 KO zebrafish. This is likely because serotonin regulates the adrenaline and cortisol levels in stressed fish [60]. Acetylcholine is essential for central nervous system functions, such as attention, learning, memory, and sleep. The dysregulation of acetylcholine can cause severe distress and is linked to conditions like anxiety, attention deficit hyperactivity disorder (ADHD), and schizophrenia. In Yu-Nung Lin et al.’s [39] study, although the brain acetylcholine levels in LPAR3 KO zebrafish did not show statistically significant changes, they were lower than those in the control group, potentially contributing to anxiety-like behavior.

It is noteworthy that the absence of the LPA1 receptor does not influence the expression or consolidation of fear 24 h after fear conditioning [29], suggesting that the LPA1 receptor may not be the primary mediator in this emotional process.

Anhedonia, which is generally characterized by a reduced ability to feel pleasure, is a fundamental symptom of mood disorders [61,62]. Moreno-Fernández RD et al. [33] observed that LPA1-null mice exhibited anhedonia, while antidepressant treatment enhanced their capacity to experience pleasure. Furthermore, in rodents, social communication primarily occurs through olfaction [63], and the duration of time that male rodents spend sniffing female urine can be used to assess pleasure-seeking behavior. The authors observed a reduction in this duration in LPA1-null mice, suggesting that the diminished spontaneous olfactory response to opposite-sex urinary pheromones in these mice might indicate both anhedonia and impaired appetitive behavior. Besides the loss of pleasure, decreased energy or increased fatigue is a core symptom of depression [64]. Moreno-Fernández RD et al. [33] modeled the decreased energy or increased fatigue of depression using nest-building behavior in mice, discovering that LPA1-null mice exhibited significantly impaired nest-building behavior, which was restored following antidepressant treatment. The study further noted that LPA1-null mice exhibited a reduced immobility time and increased climbing behavior in the behavioral despair test, suggesting an abnormal emotional response to stress. Given that anhedonia and heightened stress reactivity are critical endophenotypes in the psychopathology of major depressive disorder [61], alterations in the LPA1 receptor may contribute to the susceptibility to comorbid depression and anxiety.

In humans, polymorphisms in the LPA1 receptor are linked to an increased risk of primary hypertension. Considering that stress is a significant environmental factor contributing to depression and increasing the susceptibility in individuals with risk alleles [65], genetic variations in this receptor might influence the pathogenesis of depression.

Despite the heterogeneity of syndromes with various comorbidities and the limitations of animal models in replicating key symptoms of human depression (such as guilt, suicidal tendencies, and profound sadness), the use of animal models remains indispensable in exploring the neurobiology of mental disorders [66]. Therefore, drugs targeting the LPA1 receptor signaling pathway may assist in regulating mood and anxiety. Investigating LPA signaling through the LPA1 receptor for the treatment of anxiety disorders holds significant promise for future therapeutic strategies.

The study by Moreno-Fernández RD et al. [35] observed that the behavioral outcomes and brain functional activity of LPA1-null mice and those treated with the LPA1 antagonist Ki16425 were inconsistent. The results indicated that the antagonist replicated some, but not all, of the effects of LPA1 receptor gene deletion. This discrepancy can be explained in several ways. Firstly, the LPA1-null mice may have developed adaptive changes in other areas, necessitating further research to analyze the differences between long-term drug-administered mice and LPA1-null mice, possibly due to the acute administration model used in the study. Secondly, systemic LPA1 receptor deficiency may have a greater effect than the brain-specific inhibition of LPA1 through Ki16425 injection. Finally, the possibility that Ki16425 lacks selectivity for LPA1 cannot be excluded.

In the study conducted by Cristina Rosell-Valle et al. [42], GABAergic precursor cells were transplanted into the adult hippocampi of both LPA1-null and wild-type mice. The results indicated a difference in the survival rates of the transplanted cells between the two genotypes, with a higher survival rate in the LPA1-null mice. A possible explanation for this outcome is that the hippocampi of LPA1-null mice, which exhibit defects in the GABAergic system, may have a reduced cell density, thereby accommodating more transplanted interneurons. This hypothesis is consistent with previously documented findings [67,68,69] and supports the concept that grafted cells may establish a conducive environment for subsequent endogenous repair processes or the formation of alternative neuronal networks to compensate for the lost interneurons’ functions [70].

Estela Castilla-Ortega et al. [30] observed that the anxiety-inducing effects of LPA were confined to highly stressful and unfamiliar environments, as LPA did not induce anxiety-like behavior in animals that had been previously acclimated to the test. When exposed to a novel environment, animals elicit strong behavioral and stress responses, which significantly diminish as the environment becomes familiar and the perceived threat reduces [71,72]. Consequently, the anxiety-inducing effects of LPA are confined to more stressful and unfamiliar situations.

Cristina Rosell-Valle et al. [41] noted that the chronic ICV injection of 18:1 LPA increased the exploratory behavior and reduced anxious tendencies in mice. However, previous research has shown that an acute intracerebroventricular (ICV) injection of 18:1 LPA led to a decrease in locomotor activity in the open field test (OF), elicited anxiety-like behaviors in the elevated plus maze, impaired novel object preference in the Y-maze test, and increased immobility in the forced swimming test [30]. Similarly, another study found that an acute ICV infusion of 18:1 LPA increased anxiety-like behavior in mice in both the elevated plus maze and hole board tests [31]. These findings, which are inconsistent with the current experiment, are likely attributable to the different application methods and dosing regimens. In contrast to the concentration used in the present study, the previous research by Estela Castilla-Ortega et al. [30] and Misa Yamada et al. [31] employed relatively higher concentrations of LPA, which could lead to receptor internalization [73,74] or the activation of other LPA receptors. Another reason could be that this experiment used a chronic continuous (21-day) administration model, which differed from previous ones. This highlights the need for further research to determine the underlying mechanisms. The continuous infusion of C18:1 LPA may enhance the integration of newly formed neurons into the hippocampal circuitry, thereby “fine-tuning” this process and improving overall function.

Sara Tabbai et al. [37] found a connection between different LPA species and behavioral effects, discovering that the 18:1 LPA levels were correlated with fear responses. However, it is noteworthy that another study evaluated the role of 18:1 LPA in behavior, showing that injecting rats with 18:1 LPA led to reduced locomotion in the open field test [30]. Although the findings from Sara Tabbai et al. [37] do not completely align with this observation, it is plausible to speculate that the concentration of 18:1 LPA may play a vital role in regulating locomotion and heightened emotional arousal. Therefore, further research is required to clarify this connection.

The synthesis of LPA is significantly influenced by the LPA3 receptor [75]. The LPA2 receptor may also contribute to LPA production, albeit to a lesser extent [76]. However, in the hippocampus, the LPA1 receptor does not appear to play a critical role in LPA synthesis. Sara Tabbai et al. [37] observed a significant increase in saturated 18:0 LPA levels in the hippocampi of LPA1-null mice. A plausible hypothesis for this observation is that the absence of the LPA1 receptor may alter the catalytic activity of phospholipase A1 (PLA1) and phospholipase A2 (PLA2), the enzymes responsible for generating saturated LPA species [12].

Considering that LPA protects brain cells from ethanol-induced alterations [77] and chronic ethanol exposure alters the levels of LPA species in mouse serum and peripheral tissue [78], current evidence indicates that LPA signaling is significantly involved in alcohol abuse behavior [32]. To further substantiate this hypothesis, additional research is needed on the various LPA species and the expression of LPA receptors in individuals exhibiting alcohol abuse.

Previous research has shown that individuals with depression have reduced plasma 5-HT levels compared to healthy controls [23]. Supporting this, patients experiencing depression during alcohol withdrawal also exhibit lower plasma levels of 5-HT and norepinephrine [24]. Furthermore, research has shown that the levels of 5-hydroxyindoleacetic acid, a metabolite of 5-HT, are reduced in the cerebrospinal fluid of individuals with alcoholism, compared to non-alcoholic counterparts of a similar age and health status [79,80,81]. These findings suggest that alcohol affects the levels and turnover of 5-HT in both the plasma and the brain [82]. Thus, the increase in the plasma 5-HT levels mediated by gintonin [34] may contribute to reducing depressive-like behaviors in alcohol-withdrawn mice. Nevertheless, previous research suggests that the systemic administration of gintonin triggers the adrenal glands to release catecholamines (such as dopamine and norepinephrine) [83]. Additionally, gintonin has been shown to promote glutamate release and enhance synaptic transmission in the hippocampus in both in vitro and in vivo studies [84]. Additionally, gintonin stimulates the release of acetylcholine and elevates the choline acetyltransferase levels in hippocampal neural progenitor cells [84]. Therefore, it is possible that the regulation of catecholamine release in the peripheral system and the modulation of glutamate and acetylcholine release in the central nervous system by gintonin also contributed to the observed alleviation of depressive-like behaviors in this study.

Xiang Nie et al. [85] reported an increase in microglial activation and elevated levels of inflammatory cytokines in the brains of mice exhibiting depressive-like behaviors. Astrocyte proliferation and microglial activation are linked to depression, neural injury, and impaired neurogenesis [86]. The activation of microglia in the prefrontal cortex and hippocampus correlates positively with depressive symptoms. SSRIs and SNRIs achieve their antidepressant effects by suppressing microglial activation [87]. Furthermore, inflammatory cytokines induce neural injury and impair neurogenesis, leading to depressive-like behaviors, while the inactivation of the Bax gene in neural progenitor cells improves neurogenesis and exhibits antidepressant-like effects [88]. These findings indicate that astrocyte proliferation, microglial activation, elevated levels of inflammatory cytokines, and impaired neurogenesis all contribute to the development of depression. MRL/lpr mice show microglial activation, astrocyte proliferation in the hippocampus, and neuronal apoptosis [89]. MRL/lpr mice are a valuable animal model for the investigation of NPSLE, as they display depression-like behaviors. The study by Wataru Nagata et al. [45] demonstrated that LPA treatment may alleviate depressive symptoms in NPSLE patients. Nevertheless, further research is needed to ascertain whether microglial activation and astrocyte proliferation in MRL/lpr mouse brains contribute to behavioral abnormalities by inducing inflammatory cytokines and whether the alleviation of these abnormalities with LPA treatment results from the inhibition of neuroinflammation.

Clinical studies suggest that TCAs may surpass other antidepressant categories in effectiveness. For instance, a network meta-analysis of 21 antidepressants for the treatment of acute major depressive disorder identified amitriptyline as the most effective option [90]. Another meta-analysis similarly revealed that tricyclic antidepressants outperformed selective serotonin reuptake inhibitors (SSRIs) in treating major depressive disorder [91]. Additionally, tricyclic antidepressants are more likely than other antidepressant types to cause patients with bipolar depression to switch from depressive to manic states [92]. The role of the LPA1 receptor might partially explain the high efficacy of TCAs. The study by Naoto Kajitani et al. [46] sheds light on the mechanism of action of TCAs, indicating that targeting the LPA1 receptor could be a promising approach for the development of more potent antidepressants.

In human studies on MDD patients, the levels of LPA have shown varying results across different research efforts. Eun Young Kim et al. [36] identified that specific species of LPA and triglycerides (TG) could differentiate between current MDD patients and healthy controls. However, the studies by Leo Gotoh et al. [38] and Sumaia Riya et al. [40] did not find significant differences in the LPA levels between MDD patients and healthy controls, nor were these levels correlated with the severity of depressive symptoms. Conversely, Wataru Omori et al. [43] discovered significantly reduced levels of LPA 22:6 in the cerebrospinal fluid of MDD patients, which negatively correlated with certain clinical symptom scores. These findings indicate that while the precise role of LPA in depression remains unclear, LPA 22:6 may have potential implications in the pathophysiology of MDD, warranting further investigation to confirm its relevance.

Although we strictly adhered to the PRISMA guidelines, the limited number of included references may have led to significant heterogeneity in the results. The experimental protocols and timeframes of the included studies were not consistent, and the intervention environments varied, potentially introducing other influencing factors.

In recent decades, research on behavior regulation has largely overlooked the role of lipids. However, increasing attention is being given to lipids due to their involvement in the pathogenesis of neurological disorders. Recent advancements in lipidomics technology now enable the accurate detection, identification, and analysis of lipid species in blood and tissue [93]. This enables researchers to investigate how the lipid composition impacts behavior [94], focusing particularly on the regulation of stress and emotional responses. Nevertheless, studies on the mechanisms by which lipids regulate emotions remain scarce. This highlights the considerable potential for advancement in this field, particularly regarding LPA. Research on LPA receptors 3 and 5 remains underexplored, and studies on LPA1 require further enhancement and completion.

## 5. Conclusions

In conclusion, research on the role of lipids in emotional regulation offers new avenues for the management of abnormal emotional behaviors and the treatment of related diseases. As research advances, incorporating validated drugs targeting the LPA/LPA receptor system into standard clinical practice could significantly transform the treatment of abnormal emotional states. However, ongoing efforts are necessary to overcome the challenges and ensure that promising therapeutic pathways for emotional disorders transition successfully from the research laboratory to clinical practice.

## Figures and Tables

**Table 1:** Database retrieval strategy.

ID	Search Terms	Results
#1	(lysophosphatidic acid[Title/Abstract]) AND (mood[Title/Abstract])	32
#2	(lysophosphatidic acid[Title/Abstract]) AND (emotion[Title/Abstract])	12
#3	(lysophosphatidic acid[Title/Abstract]) AND (depression[Title/Abstract])	99
#4	(lysophosphatidic acid[Title/Abstract]) AND (anxiety[Title/Abstract])	65

**Table 2:** Results of quality evaluation of included literature.

Study	Risk of Bias				Applicability Concerns		
	Patient Selection	Index Text	Reference Standard	Flow and Timing	Patient Selection	Index Text	Reference Standard
L.J. Santin et al. [26]	Low risk	Low risk	Low risk	Unclear risk	Low risk	Low risk	Low risk
Estela Castilla-Ortega et al. [27]	Low risk	Low risk	Low risk	Unclear risk	Low risk	Low risk	Low risk
Z. Callaerts-Vegh et al. [28]	Low risk	Low risk	Low risk	Unclear risk	Low risk	Low risk	Low risk
C. Pedraza et al. [29]	Low risk	Low risk	Low risk	Unclear risk	Low risk	Low risk	Low risk
Estela Castilla-Ortega et al. [30]	Low risk	Low risk	Low risk	Unclear risk	Low risk	Low risk	Low risk
Misa Yamada et al. [31]	Low risk	Low risk	Low risk	Unclear risk	Low risk	Low risk	Low risk
Estela Castilla-Ortega et al. [32]	Low risk	Low risk	Low risk	Unclear risk	Low risk	Low risk	Low risk
Moreno-Fernández RD et al. [33]	Low risk	Low risk	Low risk	Unclear risk	Low risk	Low risk	Low risk
Hyeon-Joong Kim et al. [34]	Low risk	Low risk	Low risk	Unclear risk	Low risk	Low risk	Low risk
Moreno-Fernández RD et al. [35]	Low risk	Low risk	Low risk	Unclear risk	Low risk	Low risk	Low risk
Eun Young Kim et al. [36]	Low risk	Low risk	Low risk	Unclear risk	Low risk	Low risk	Low risk
Sara Tabbai et al. [37]	Low risk	Low risk	Low risk	Unclear risk	Low risk	Low risk	Low risk
Leo Gotoh et al. [38]	Low risk	Low risk	Low risk	Unclear risk	Low risk	Low risk	Low risk
Yu-Nung Lin et al. [39]	Unclear risk	Low risk	Low risk	Unclear risk	Low risk	Low risk	Low risk
Sumaia Riya et al. [40]	Low risk	Low risk	Low risk	Unclear risk	Low risk	Low risk	Low risk
Cristina Rosell-Valle et al. [41]	Low risk	Low risk	Low risk	Unclear risk	Low risk	Low risk	Low risk
Cristina Rosell-Valle et al. [42]	Low risk	Low risk	Low risk	Unclear risk	Low risk	Low risk	Low risk
Wataru Omori et al. [43]	Low risk	Low risk	Low risk	Unclear risk	Low risk	Low risk	Low risk
Moreno-Fernández RD et al. [44]	Low risk	High risk	Unclear risk	Unclear risk	Low risk	High risk	Unclear risk
Wataru Nagata et al. [45]	Low risk	Low risk	Low risk	Unclear risk	Low risk	Low risk	Low risk
Naoto Kajitani et al. [46]	Low risk	Low risk	Low risk	Unclear risk	Low risk	Low risk	Low risk

**Table 3:** Review of selected preclinical research literature.

Authors	Year	Group	Intervention	Results
L.J. Santin et al. [26]	2009	LPA1-null mice	unknown	anxiety
Estela Castilla-Ortega et al. [27]	2010	LPA1-null mice	unknown	anxiety
Z. Callaerts-Vegh et al. [28]	2012	LPA5-null mice	unknown	significantly reduced anxiety levels
C. Pedraza et al. [29]	2013	LPA1-null mice	unknown	fear extinction impairment
		wild-type mice	LPA1 antagonist Ki16425 administration	fear extinction impairment
Estela Castilla-Ortega et al. [30]	2014	rats	LPA administration	anxiety and depression
Misa Yamada et al. [31]	2015	adult mouse	LPA administration	anxiety
Estela Castilla-Ortega et al. [32]	2016	LPA1-null mice	alcohol withdrawal	anxiety
		wild-type mice	alcohol withdrawal + LPA1 antagonist Ki16425 administration	increased ethanol consumption
Moreno-Fernández RD et al. [33]	2017	LPA1-null mice	unknown	panic-like responses, anhedonia, restlessness, and increased stress reactivity
		LPA1-null mice	antidepressant treatment	depressive-like behaviors improved
Hyeon-Joong Kim et al. [34]	2017	enterochromaffin cell line BON cells	gintonin administration	release of 5-hydroxytryptamine
		C57BL/6 mice	gintonin administration	depressive-like behaviors improved
Moreno-Fernández RD et al. [35]	2018	LPA1-null mice	unknown	anxiety and depression
Sara Tabbai et al. [37]	2019	LPA1-null mice	unknown	marked rise in the levels of saturated 18:0 LPA in the hippocampus
		mice	acute stress	changes in hippocampal LPA levels
Yu-Nung Lin et al. [39]	2020	LPAR3 knockout (KO) zebrafish	unknown	anxiety
Cristina Rosell-Valle et al. [41]	2021	mice	21-day sustained intracerebroventricular (ICV) infusion of C18:1 LPA and LPA1-3 receptor antagonist Ki16425	reduced anxious tendencies
Cristina Rosell-Valle et al. [42]	2021	LPA1-null mice	unknown	hippocampal GABAergic system defects
		LPA1-null mice	GABAergic precursor cell transplantation	normalize or mitigate hippocampal GABAergic system defects
Moreno-Fernández RD et al. [44]	2023	LPA1-null mice	DEX	diminished response to DEX
Wataru Nagata et al. [45]	2023	MRL/lpr mice	LPA administration	alleviates depressive-like behaviors
Naoto Kajitani et al. [46]	2023	LPA1 receptor	tricyclic and tetracyclic antidepressant administration	TCAs directly bind to LPA1 receptor and exhibit G-protein-biased agonism

LPA: lysophosphatidic acid; DEX: dexamethasone suppression test.

**Table 4:** Review of selected clinical research literature.

Authors	Year	Group	Intervention	Results
Eun Young Kim et al. [36]	2018	patients with current MDD (cMDD)	unknown	LPA (16:1), TG (44:0), and TG (54:8) differentiated cMDD from healthy controls with 76% accuracy
Leo Gotoh et al. [38]	2019	patients with MDD	unknown	no significant differences in LPA levels between MDD patients and healthy controls in either CSF or plasma samples
Sumaia Riya et al. [40]	2020	patients with MDD	unknown	no significant differences in serum LPA and LPC levels between MDD patients and healthy controls
Wataru Omori et al. [43]	2021	patients with MDD and SCZ	unknown	significantly lower levels of LPA 22:6 in CSF of patients with MDD and SCZ compared to healthy controls

LPA: lysophosphatidic acid; MDD: major depressive disorder; CSF: cerebrospinal fluid; SCZ: schizophrenia.

## Supplementary Materials

The following supporting information can be downloaded at: https://www.mdpi.com/article/10.3390/ijms25137440/s1. Reference [95] is cited in the Supplementary Material.
